# Cinacalcet use and the risk of cardiovascular events, fractures and mortality in chronic kidney disease patients with secondary hyperparathyroidism

**DOI:** 10.1038/s41598-018-20552-5

**Published:** 2018-02-01

**Authors:** Marie Evans, Shona Methven, Alessandro Gasparini, Peter Barany, Kate Birnie, Stephanie MacNeill, Margaret T. May, Fergus J. Caskey, Juan-Jesus Carrero

**Affiliations:** 1 United Kingdom Renal Registry (UKRR), Southmead Hospital, Bristol, BS10 5NB UK; 2 Division of Renal Medicine, Department CLINTEC, Karolinska Institutet, Stockholm, Sweden; 3 School of Social and Community Medicine, University of Bristol, Bristol, BS8 2PS United Kingdom; 4 Department of medical epidemiology and biostatistics, Karolinska Institutet, Stockholm, Sweden; 5 Department of Health Sciences, University of Leicester, Leicester, United Kingdom

## Abstract

With the aim to expand the randomized controlled trial evidence of cinacalcet treatment to the unselected, general chronic kidney disease (CKD) population we analysed a large inception cohort of CKD patients in the region of Stockholm, Sweden 2006–2012 (both non-dialysis, dialysis and transplanted) with evidence of secondary hyperparathyroidism (SHPT). We used marginal structural models to account for both confounding by indication and time-dependent confounding. Over 37 months, 435/3,526 (12%) initiated cinacalcet *de novo*. Before cinacalcet initiation, parathyroid hormone (PTH) had increased progressively to a median of 636ng/L. After cinacalcet initiation, PTH declined, as did serum calcium and phosphate. In total, 42% of patients experienced a fatal/non-fatal cardiovascular event, 32% died and 9% had a new fracture. The unadjusted cardiovascular odds ratio (OR) associated with cinacalcet treatment was 1.01 (95% confidence interval: 0.83, 1.22). In the fully weighted model, the cardiovascular odds was lower in cinacalcet treated patients (OR 0.67: 0.48, 0.93). The adjusted ORs for all-cause mortality and for fractures were 0.79 (0.56, 1.11) and 1.08 (0.59, 1.98) respectively. Our study suggests cinacalcet treatment improves biochemical abnormalities in the wider CKD population, and adds real-world support that treating SHPT with cinacalcet may have beneficial effects on cardiovascular outcomes.

## Introduction

Chronic kidney disease (CKD) is a common disease with a life-time risk in the United States as high as 59%^1^. Those affected by CKD have an increased risk of cardiovascular disease and death already from early CKD stages^2^. The increased levels of parathyroid hormone (PTH), often seen in patients with CKD, has been associated with the increased risk of cardiovascular events, fractures and mortality^3^. In those with end-stage kidney disease, secondary hyperparathyroidism (SHPT) accelerates vascular calcification and subclinical atherosclerosis^4,5^ and further lead to hyper-dynamic bone disease and osteitis fibrosis cystica (renal osteodystrophy).

Cinacalcet acts on the calcium-sensing receptor and effectively decreases parathyroid hormone (PTH) levels^6^. In addition, cinacalcet can also improve phosphate balance and reduce hypercalcemia^7,8^. Furthermore, the combined treatment with cinacalcet and vitamin D delays the progression of valvular, aortic and coronary calcification score^9^. Yet, these favourable changes in biochemical and proxy markers have not been followed by significant reductions in hard clinical outcomes. The Evaluation Of Cinacalcet hydrochloride therapy to Lower cardiovascular events (EVOLVE) trial was inconclusive and failed to show any statistically significant effect on its primary outcome, a composite of cardiovascular events and death^10^. However, predefined secondary and post-hoc analyses of the EVOLVE trial suggested beneficial effects both in general and in subgroups. As a consequence of these mixed results, it has been proposed that whilst cinacalcet could be used as one of several drugs to improve the achievement of biochemical control in chronic kidney disease related mineral bone disorder as recommended by international guidelines^11^, it should not to be used with the purpose of reducing cardiovascular outcomes and/or improving survival^12^.

As with several other randomized controlled trials in nephrology which later showed negative results, the EVOLVE study was preceded by observational data indicating a positive effect of the treatment^13,14^. However,, although studies using traditional statistical models could adjust for confounding by indication they will inherently suffer from time-dependent confounding. In observational studies, treatments are often given more frequently to individuals with severe illness who have a higher mortality risk. Consequently, we need to adjust for differences between treated and non-treated. We also have to consider that people who initiate treatment are more likely to be those with a progressive disease. Traditional methods allow adjustment for time-updated measurements, but do not take into account that the current level of the risk factor also is influenced by previous decisions to treat or not to treat. In the example of cinacalcet, PTH level is both a determinator of the risk of treatment and a risk factor for death. Also, the current PTH level is affected by treatment/non-treatment. Studies of the association between cinacalcet and outcome therefore require statistical models which consider time-dependent confounding.

Marginal structural models (MSM) are advanced statistical models which adjust for both baseline confounding by indication and time-dependent confounding. The MSM approach result in less biased results if applied to cohorts where rich information on characteristics used to allocate treatment status and predict outcomes are available in repeated measurements over time. Carefully performed observational studies have proved to be a useful adjunct to randomized trials in order to translate and reproduce their results in wider and more heterogeneous populations^15,16^. There are now numerous examples where MSMs have been used to guide clinical decision-making; most of them are connected to HIV research^17,18^. In this study we aimed to analyse if treatment with cinacalcet in CKD patients with SHPT was associated with improved cardiovascular outcomes, all-cause mortality or fractures compared with non-treatment. We undertook this study in a complete healthcare utilization cohort in Stockholm, Sweden using MSMs^19^ to provide less biased estimates by controlling for baseline confounding by indication and time-dependent confounding.

## Results

There were 3,526 adult patients with CKD in the region of Stockholm known to specialist renal services who had a parathyroid hormone (PTH) twice above the upper reference range during the study inclusion period. The median age was 66.8 years and 64.6% were men (Web Table 7). The median PTH was 183 ng/L (interquartile range [IQR] 150–265) and estimated glomerular filtration rate was 18.6 ml/min/1.73 m^2^ (IQR 9.4–27.1) at inclusion. At index date, 607 (17.2%) of the patients were receiving dialysis, and 415 (11.8%) had received a renal transplant. The baseline characteristics in relation to the EVOLVE study cohort are presented in the Web Table 8.

### Cinacalcet treatment

During a median follow-up of 37 months (IQR 18–61), 435 (12.3%) patients initiated treatment with cinacalcet. The patients who were prescribed cinacalcet were younger (60.8 versus 67.7 years among the non-users), more often had a history of cardiovascular disease, and were more often in dialysis (Table 1). Patients who were subsequently prescribed cinacalcet had higher PTH, phosphate and calcium levels than non-users. They were more users of active vitamin D, erythropoiesis stimulating agents, calcium supplements, and phosphate binders at inclusion but less often users of angiotensin converting enzyme inhibitors and beta-blockers.

**Table 1:** Baseline Characteristics of the Chronic Kidney Disease Cohort According to Received Cinacalcet Treatment, Stockholm, Sweden 2006–2011.

		No Cinacalcet (n = 3091)	New Cinacalcet use (n = 435)	P-value
Characteristic	Age, median years	67.7 (56–76)	60.8 (50–70)	<0.001
	Men	2025 (65.5)	252 (57.9)	0.01
	Women	1066 (34.5)	183 (42.1)	
Comorbidity	Hypertension	1427 (46.2)	178 (40.9)	0.08
	Cardiovascular disease	1338 (43.3)	142 (32.6)	<0.001
	Diabetes mellitus	675 (21.8)	86 (19.8)	0.48
	Charlson comorbidity index, mean	3.96 (2.2)	3.68 (1.9)	0.11
	Previous surgery for hyperparathyroidism	44 (1.4)	6 (1.4)	0.98
	Previous fracture	400 (12.9)	49 (11.3)	0.57
Laboratory	Parathyroid hormone, median	177 (147–243)	316 (202–548)	<0.001
	P-Phosphate, median	1.3 (1.1–1.6)	1.6 (1.3–2.0)	<0.001
	B-Haemoglobin	119.8 (17.1)	119.9 (15.4)	0.03
	P-Albumin, median	35 (32–38)	35 (32–38)	0.03
	P-Calcium, median	2.25 (2.17–2.34)	2.37 (2.25–2.50)	<0.001
	P-Creatinine, median	256 (191–394)	499 (286–739)	<0.001
	Albuminuria no	640 (31.8)	32 (22.2)	0.006
	Albuminuria micro	367 (18.3)	21 (14.6)	
	Albuminuria macro	1,006 (50.0)	91 (63.2)	
Medication	ACEi or ARB	2237 (72.4)	272 (62.5)	0.001
	Beta-blocker	2009 (65.0)	248 (57.0)	0.007
	Vitamin D (active)	1,396 (45.2)	240 (55.2)	0.001
	Vitamin D nutritional	193 (6.2)	13 (3.0)	0.27
	Erythropoiesis stimul. agents	936 (30.3)	185 (42.5)	0.001
	Calcium supplement	961 (31.1)	171 (39.3)	0.005
	Phosphate binder	320 (10.4)	156 (35.9)	<0.001
	Prednisolone	790 (25.6)	86 (19.8)	0.050
RRT	Dialysis	417 (13.5)	190 (43.7)	<0.001
	Renal transplantation	363 (11.7)	52 (12.0)	0.94
Outcomes				
	Cardiovascular event	1295 (41.9)	203 (46.7)	0.11
	Hospitalization ischemic heart disease/myocardial infarction	502 (16.2)	43 (9.9)	0.19
	Hospitalization heart failure	523 (16.9)	40 (9.2)	0.08
	Hospitalization stroke	136 (4.4)	11 (2,5)	0.67
	Hospitalization other cardiovascular	119 (3.8)	22 (5.1)	0.48
	Cardiovascular death	15 (0.5)	87 (20.0)	0.02
	All-cause mortality	993 (32.1)	137 (31.5)	0.79
	Fracture	383 (11.9)	52 (16.8)	0.22

The variables are presented as number (percentages) if categorical and median (interquartile range, IQR) or mean (Standard deviation, SD) if continuous. ACEi (Angiotensin Converting Enzyme inhibitor), ARB (Angiotensin Receptor Blocker). eGFR (glomerular filtration rate estimated by CKD-EPI equation) in ml/min/1.73 m^2^, RRT (renal replacement therapy), P-values estimated by Kruskall-Wallis (Chi2) for categorical variables and Pearson Chi2 for continuous variables.

**Figure 1:**
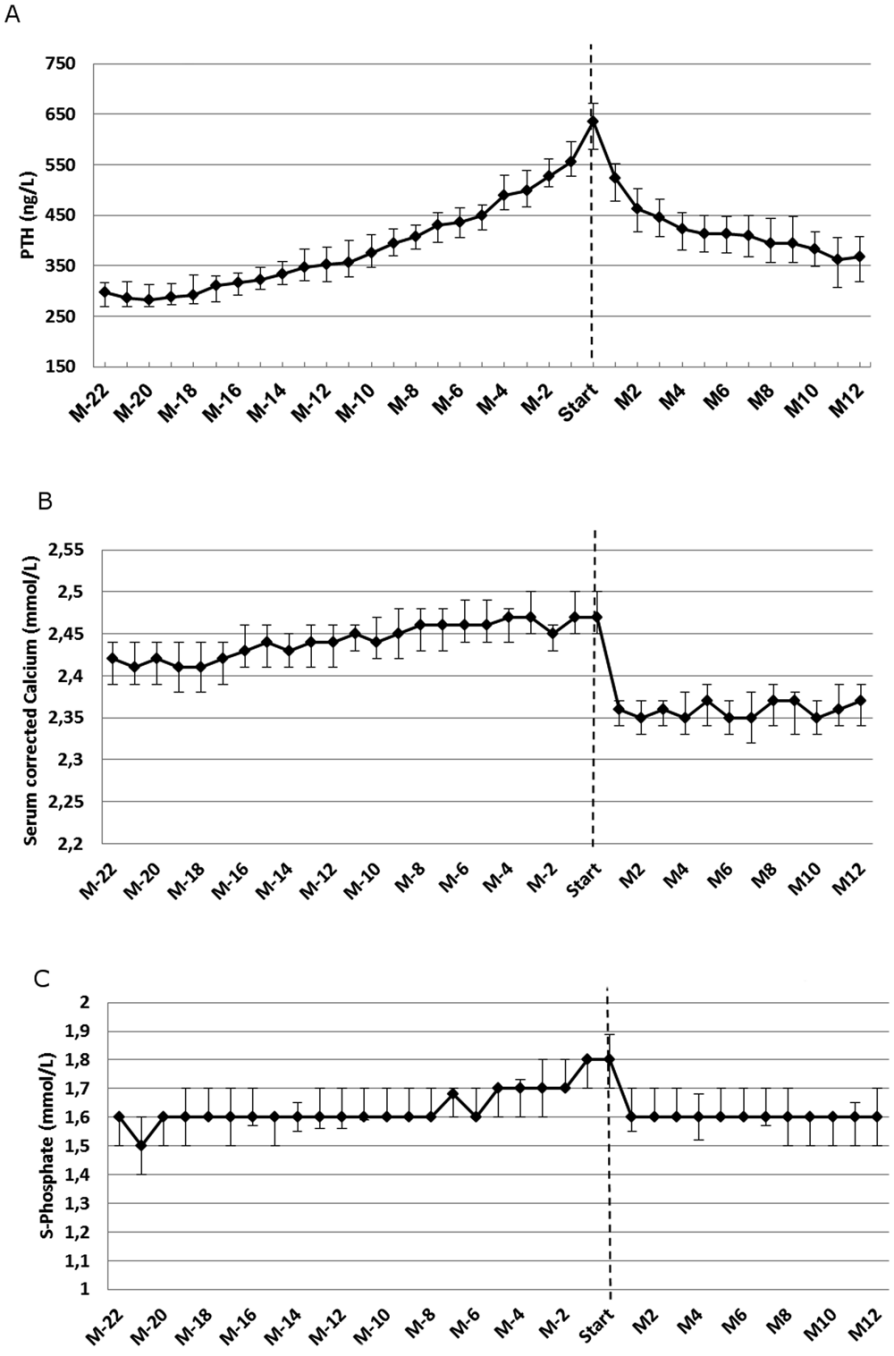
Development of parathyroid hormone (**A**), corrected calcium (**B**) and phosphate (**C**) among referred CKD patients in Stockholm, Sweden 2006–2012 before and after Cinacalcet initiation. Below figure: Parathyroid hormone levels (PTH) in ng/L with 95% confidence interval. M(month). Negative signs indicate that the month is prior to treatment initiation and positive numbers that the month is after the treatment initiation.

Treatment with cinacalcet was initiated after a median of 19 months from study inclusion. By the time cinacalcet was started, the median PTH had risen to 636ng/L. Most patients were on dialysis (n = 272; 62.5%) or had a transplant (n = 54; 12.4%), though a substantial proportion (n = 109; 25.1%) were pre-dialysis patients with a mean eGFR of 14 ml/min/1.73 m^2^ (Table 2). Three and twelve months after initiating cinacalcet, the median PTH had dropped to 446 ng/L (95% Confidence interval: 408, 482) and 368 ng/L (95% CI: 318, 408), respectively (Fig. 1, panels A–C). Serum calcium values increased slightly in the period preceding cinacalcet initiation, although they remained within the normal range even just before cinacalcet treatment started. After cinacalcet initiation, calcium dropped abruptly to somewhat lower values. Serum phosphate stayed rather constant during follow-up but increased from values just above the normal range to slightly higher values about six months before cinacalcet was started. Once cinacalcet was initiated, phosphate also dropped.

**Table 2:** Characteristics at the Time of Cinacalcet initiation in Chronic Kidney Disease Patients, Stockholm, Sweden 2006–2012.

		At Cinacalcet initiation (n = 435)
Timing of cinacalcet initiation	Months after baseline, mean (SD)	22.8 (17.0)
Comorbidity before initiation	Charlson comorbidity index	3.68 (1.9)
	New fracture before cinacalcet initiation	18 (4.1)
	New cardiovascular event before cinacalcet initiation	76 (17.5)
Laboratory values	Parathyroid hormone, median (IQR)	636 (436–860)
	P-Phosphate, median (IQR)	1.8 (1.4–2.2)
	B-Haemoglobin	119.5 (15.1)
	P-Albumin	34.0 (4.3)
	P-Calcium, median (IQR)	2.45 (2.28–2.59)
	Albuminuria (%)	
	none	30 (12.0)
	micro	43 (17.1)
	macro	178 (70.9)
Medication	Use of ACEi or ARB	283 (65.1)
	Use of Beta-blocker	284 (65.3)
	Use of Vitamin D (active)	316 (72.6)
	Use of Vitamin D supplement	14 (3.2)
	Use of erythropoiesis stimulating agents	212 (48.7)
	Use of Calcium supplement	196 (45.1)
	Use of Phosphate binder	316 (72.6)
	Use of Prednisolone	100 (23.0)
Renal replacement therapy (RRT)	No RRT	109 (25.1)
	Dialysis	272 (62.5)
	Renal transplantation	54 (12.4)

The variables are presented as number (percentages) if categorical and median (interquartile range, IQR) or mean (Standard deviation, SD) if continuous. eGFR (glomerular filtration rate estimated by CKD-EPI equation) in ml/min/1.73 m^2^, ACEi (Angiotensin Converting Enzyme inhibitor), ARB (Angiotensin Receptor Blocker).

### Cardiovascular event and all-cause mortality

In total, 1,498 patients (42.3%) experienced the composite primary outcome (a new cardiovascular event) during follow-up resulting in a cardiovascular event rate of 14.9/100 patient-years. Hospitalizations for new cardiovascular events were attributed to heart failure (n = 563; 37.6%), followed by ischemic heart disease/myocardial infarction (n = 545; 36.4%), and cerebrovascular disease (n = 147; 9.8%) Only 102 patients (6.8%) died from cardiovascular death with no prior cardiovascular event. In total 1,130 patients (32.1%) died. Cardiovascular disease was the most common cause of death (n = 453; 40.1%), followed by metabolic disease (renal or endocrinological) (n = 354; 31.3%), cancer (n = 112; 9.9%) and infection (n = 69; 6.1%). The overall mortality rate was 9.6/100 person-years.

In the primary analysis of cardiovascular event risk, the unadjusted odds ratio (OR) for cinacalcet use versus non-use was 1.01 (0.82, 1.22) (Web Figure 1, Table 3). The model adjusted using inverse probability of treatment and censoring weights displayed lower cardiovascular odds among cinacalcet users (OR 0.67: 95% CI: 0.48, 0.93). The cause-specific ORs were rather similar for ischemic heart disease, heart failure and stroke (Subgroup analysis in Web appendix). The unadjusted OR of all-cause mortality associated with cinacalcet use was 0.98 (95% CI: 0.82, 1.19). The fully weighted OR for all-cause mortality associated with cinacalcet use was 0.79; 95% CI: 0.56, 1.11. There was no evidence of effect modification (P > 0.1) by age strata, gender, presence of diabetes mellitus, or dialysis status and therefore stratified analyses were not estimated (Web Appendix). Sensitivity analyses did not meaningfully modify the magnitude or the direction of the associations observed (Web Tables 1–7). The results were consistent regardless of whether we used truncation of the treatment weights at 1,99^th^ percentile, at treatment weights >10, excluded patients who had the highest weights in the model, analysed only incident SHPT patients, included baseline blood pressure data, or analysed time on treatment.

**Table 3:** Odds of Outcomes Associated with Cinacalcet Treatment in Chronic Kidney Disease Patients, Stockholm, Sweden 2006–2012.

	Crude OR (n = 3526)	Model 1* (n = 3339)	Model 2 ^**^ (main)(n = 3339)	Model 3^#^ (n = 3339)
Cardiovascular event	1.01 (0.83–1.22)	0.52 (0.34–0.81)	0.67 (0.48–0.93)	0.68 (0.51–0.92)
All-cause mortality	0.98 (0.82–1.19)	0.73 (0.49–1.07)	0.79 (0.56–1.11)	0.83 (0.62–1.10)
Fracture	1.15 (0.80–1.67)	1.06 (0.58–1.95)	1.08 (0.59–1.98)	1.15 (0.67–1.97)

All results presented as Odds ratios OR) with 95% confidence intervals. Only months with complete information are included.

Cardiovascular event (non-fatal or fatal myocardial infarction, cerebrovascular event, angina, congestive heart failure or peripheral arterial disease). *Fully-weighted model with no truncation of weights and adjustment for baseline variables in the MSM, **Fully-weighted model with truncation at 1,99^th^ percentile and adjustment for baseline variables in the MSM, ^#^Fully-weighted model with truncation at 1,99^th^ percentile without adjustment for baseline variables in the MSM.

The adjustment variables included in the stabilized weights (treatment and censoring weights) are presented in the Web Appendix. In short they included history of comorbid conditions, age and sex, routine laboratory variables at inclusion and monthly during follow-up, relevant medication at inclusion and monthly during follow-up, Charlson comorbidity index at inclusion and during follow-up, PTH-assay, dialysis and transplantation, and treatments 3–12 months before the current month.

### Fractures

There were 309 (8.8%) patients who experienced a new fracture during follow-up which meant a fracture rate of 2.7/100 patient-years. The majority of the fractures were hip fractures (n = 122; 39.5%), followed by fractures of the pelvis and lumbar region (n = 44; 14.2%), upper arm (n = 38; 12.3%), lower leg and knee (n = 30; 9.7%) and lower arm (n = 27; 8.7%). The unadjusted OR for fracture associated with cinacalcet use was 1.15 (95% CI: 0.80, 1.67). The estimate decreased slightly in the weighted model (OR 1.08; 95% CI: 0.59, 1.95) with no evidence of association. There was weak evidence of interaction (p = 0.09) for fracture risk with the presence of diabetes mellitus. Among people with diabetes, cinacalcet treatment was associated with a non-significantly lower odds of a new fracture (OR 0.42; 95% CI: 0.15, 1.19). The association was observed in the opposite direction for those without diabetes (OR 1.40: 0.78, 2.51). No effect modification was seen for age strata, sex or dialysis/transplantation status at baseline (Web Appendix).

## Discussion

In this analysis of referred CKD patients in the region of Stockholm with secondary hyperparathyroidism, we found that initiation of cinacalcet was associated with a lower odds of cardiovascular events compared with no use. No differences in all-cause mortality or fracture risk were observed between cinacalcet users and non-users.

Observational studies have consistently showed that markedly elevated PTH levels are associated with worse outcomes both in dialysis patients^20^ and after renal transplantation^21^. However, there is also evidence that low PTH and adynamic bone disease predispose to increased vascular calcification and higher mortality^20,22^. Such findings have raised concerns about over-suppressing PTH with cinacalcet, especially in patients with preserved renal function^23^. In our “real world” observational study, patients that initiated cinacalcet had *progressively* increasing PTH levels with a median value above the Kidney Disease improving global Outcomes (KDIGO) guideline targets at the time of initiation. After cinacalcet initiation, median PTH concentration dropped within KDIGO targets and both calcium and phosphate levels normalized.

It is noteworthy that PTH and calcium levels at initiation of cinacalcet in our study were very similar to the baseline values of EVOLVE patients^24^, but phosphate levels were slightly higher. Patients included in our study were on average 10 years older, as displayed in Web Table 9. Although they less often had diabetes mellitus or hypertension diagnosed, our patients had a higher prevalence of heart failure, myocardial infarction and stroke at the time of cinacalcet initiation. However, our patient population was more heterogeneous because we considered any new cinacalcet user in the region with CKD stage 3+ and renal replacement therapy (haemodialysis, peritoneal dialysis and renal transplantation). Therefore our results expand the population originally targeted in the randomized trial.

Although the results of the EVOLVE study did not attain statistical significance for its primary outcome, prespecified secondary analyses (using both time-on-treatment and inverse probability weighting) and post-hoc intention to treat analyses adjusting for a statistically significant difference in age between the treated and non-treated suggested a beneficial effect on cardiovascular events^10^, especially among elderly patients^24^. Furthermore, combined *post hoc* meta-analysis of four earlier randomized controlled trials indicated lower rates of hospitalization for cardiovascular events^25^. Our study agrees with these observations, and our estimates are close to those found in the meta-analysis. In contrast to EVOLVE, we did not include death from any cause in our primary end-point, but only cardiovascular death and events. We in fact show that cinacalcet users had a similar unadjusted cardiovascular event risk as the non-users, but that after careful adjustment for past and current clinical history, cincalcalcet treatment was associated with lower incidence of cardiovascular events.

The weaker association with mortality observed in our study disagrees with a previous large observational report in dialysis patients using traditional statistical methods which found a lower risk for all-cause and cardiovascular mortality associated with cinacalcet^14^. Cinacalcet use in that study was associated with lower mortality in the *unadjusted* models. We believe this can be attributed to time-dependent confounding, given that they included prevalent dialysis patients and were a subset of survivors with lower cardiovascular risk. Time-dependent confounding may also have played a role later as those predicted to live long enough to benefit from cinacalcet were preferentially selected. This bias cannot be adequately addressed by traditional statistical models, but is accounted for in our analysis by using inverse probability weights.

Our results failed to show any association between cinacalcet use and the risk of fractures. At least two previous studies have suggested lower fracture incidence among cinacalcet treated patients^25,26^. The patients in both of these preceding studies were on average about 10 years younger than our patients, and our cohort included a broad mix of patients with different stages of CKD, undergoing dialysis and renal transplantation. It is possible that the fracture risk differs between these populations, but the event rate in our study was low compared with the other two studies and the confidence intervals wide, which prevented us from performing stratified analyses. Competing risk of death may have reduced fracture incidence more in our older population. It is also possible that our prediction of treatment models worked less well for fractures, since many of the characteristics related to fracture risk such as frailty and lean body mass are not captured among diagnostic codes. Finally, PTH is a poor predictor of bone histology and there is a big overlap where patients of normal-elevated PTH values may have either increased or decreased bone turnover^27^.

In CKD-related mineral bone disorder, all the disturbances in calcium, phosphorus and PTH must be collectively considered when selecting the appropriate treatment^28^. While our study does not aim to compare different SHPT management strategies (active and native vitamin D, vitamin D analogues, phosphate lowering drugs, and parathyroidectomy) we believe that our results should be interpreted in the context of the overall improved mineral balance observed in the cinacalcet users. The association between cinacalcet use and lower cardiovascular events may have been mediated through better control of phosphate, calcium, PTH and/or all of them. In the newly updated KDIGO guidelines on CKD-related mineral bone disorder it is suggested that patients requiring PTH-lowering therapy could be treated by calcimimetics, calcitriol, vitamin D analogues or any appropriate combination of these treatments^29^. The pathophysiological mechanisms of the relationship between improvement in the mineral bone disorder axis and cardiovascular disease explain why our results favour our primary end-point more than all-cause mortality, where any positive association ought to be mediated via improved cardiovascular health and not through other mechanisms such as improvement of infection or cancer-related mortality.

There are several strengths to our study. First, due to the complete coverage of the health care utilization of our region we were able to include referred CKD Stage G3+ patients and with richness of information of unprecedented precision and virtually no loss to follow up. Cinacalcet was first introduced to the Swedish market in 2005. Since we excluded patients who were prescribed cinacalcet before 2006, prevalent patients with long-standing severe SHPT were not included, allowing us to thus construct an inception cohort. In Sweden at the time, cinacalcet was prescribed at the discretion of the treating nephrologist. Due to the poor guidelines for CKD-related mineral bone disorder during the first years of the study there were no specified recommendations on when cinacalcet could be prescribed for SHPT. Often cinacalcet was used in the region as an option before, or instead of parathyroidectomy. Cinacalcet was included in the general reimbursement for patients and thus dispensed a very low cost or no cost. Quite surprisingly, a relatively large proportion was prescribed cinacalcet off-label, already before dialysis initiation. However, due to differences in clinical practice between nephrologists and clinics in the region there was a wide variation of when and to whom the drug was prescribed. Our advanced statistical analysis made use of these differences. The EVOLVE study was not published until 2012 and it is unlikely that it would have influenced prescription patterns in our study.

As with all observational studies, there are also limitations. Some factors influencing a doctor’s decision to treat are difficult to measure, e.g. “frailty”. Thus, residual unmeasured confounding may have affected our results, but, the use of a rich dataset with all recorded laboratory values ever analysed during the time period, and extensive linkages to other healthcare sources minimized this as much as possible. However, we acknowledge for instance the lack of measurements of 25-OH-vitamin D levels or alkaline phosphatase. We also mostly lacked information on clinical measures such as blood pressure, smoking status and body mass index. However, in sensitivity analyses including patients with available blood pressure data at baseline, we found no major difference in the size or direction of the estimates.

In conclusion, in this analysis of a large, regional inception cohort of CKD stage 3+ patients with SHPT, treatment with cinacalcet was associated with lower risk of cardiovascular events. In general, those treated with cinacalcet had progressive disturbances in calcium, phosphate and PTH before treatment was started, which were improved after cinacalcet initiation. This study adds real-world support that treating SHPT with cinacalcet may have beneficial effects on cardiovascular outcomes. It suggests that the results from the EVOLVE *are* applicable to the wider, secondary care CKD population – given that generalizability is always a concern about evidence from randomized controlled trials. However, it is not possible from our study to compare cinacalcet with other treatments that may be equally effective to correct SHPT and other biochemical abnormalities.

## Methods

### Study population, exposure and measurements

This study is based on the Stockholm CREAtinine Measurements (SCREAM) project^30^, a healthcare-utilization cohort in Stockholm, Sweden between 2006–2012 described in more detail elsewhere^30^. We selected all adult (≥18 years) individuals with CKD Stage G3 + (estimated glomerular filtration rate <60 ml/min/1.73 m^2^) attending secondary care nephrology who had ever had a PTH test twice above the upper reference limit, ≥130 ng/L and who had never used cinacalcet (Web Figures 2). The study protocol was approved by the ethical committee in Stockholm. According to Swedish law, written consent is not required because quality control is an inherent element of hospital health care.

The first date of a pharmacy dispensation (ascertained by the National Registry for Dispensed drugs) of cinacalcet was used to determine date of treatment initiation. Study covariates considered included age, sex, and a variety of laboratory tests, comorbidities and medications. All consecutive laboratory tests, performed in connection with encounters in primary care, outpatient specialist care or hospital care, were extracted from the three laboratories that provide services to the region. For this study we extracted all available laboratory results for plasma PTH, creatinine, phosphate, calcium, haemoglobin, albumin, c-reactive protein, and proteinuria. PTH was measured by four different methods at the three laboratories. All three laboratories used second generation assays which also measured the PTH fragments and reported the results in ng/L. All serum creatinine measurements were standardized to isotope dilution mass spectrometry standards. Glomerular filtration rate was estimated by the CKD-EPI equation^31^.

Comorbidities and surgical procedures were identified by ICD-10 codes issued in primary care health records, specialized out-patient care, and in-hospital care. We calculated co-morbidity history at baseline and a combined Charlson comorbidity index^32^. Concurrent medication use considered all consecutive pharmacy purchases of angiotensin converting enzyme inhibitors, angiotensin receptor blockers, beta blockers, calcium supplements, non-calcium containing phosphate binders, active vitamin D, nutritional vitamin D (inactive), erythropoiesis stimulating agents, and prednisolone or other steroids (see Web Appendix for definitions, ICD-10 and ATC codes).

### Study outcomes

The primary outcome was the composite of non-fatal and fatal cardiovascular events (hospitalization or death caused by myocardial infarction, angina pectoris, congestive heart failure, cerebrovascular disease, and peripheral arterial disease). Secondary outcomes were all-cause mortality and any new fracture during follow-up.

### Statistical analysis

The data were analysed as per protocol, decided *a priori*, and based upon current knowledge of factors likely to influence clinical history and treatment decisions. Patients were followed from the first PTH twice above the reference until end of follow-up. They were regarded unexposed until the first dispensation date of cinacalcet and exposed all months thereafter (applying an intention-to-treat approach). Both time-fixed and time-dependent variables were considered. The time-fixed variables were age, sex, history of diabetes, cardiovascular disease, hypertension, and previous surgery for hyperparathyroidism at index date. Continuous time-dependent variables (haemoglobin, albumin, phosphate, creatinine, and estimated glomerular filtration rate) were categorized into quintiles of distribution. PTH assay was used as a categorical time-varying covariate for the four different types of analyses used. Medication, renal transplantation, dialysis treatment and Charlson comorbidity index were used as categorical, time-varying covariates.

We estimated the stabilized inverse probability weights of treatment monthly and accumulated until treatment initiation using a logistic regression model which modelled the association of cinacalcet use with all time-fixed and time-varying covariates described above plus a time covariate^33^. We calculated the stabilized censoring weights similarly. To reduce the risk of extreme weights affecting our results, we truncated the treatment weights at the 1,99^th^ percentile (0.096, 2.76)^33^ and used the truncated weights in all our main analyses. The variables used in the analyses, and distributions of treatment weights with and without truncation at different levels are presented in the Web Appendix. Our final weighted MSM included the stabilized treatment and censoring weights adjusting for past and current confounders of treatment. We also performed interaction tests for possible effect modification by some predetermined subgroups (age above and below 65 years, sex, history of diabetes and renal replacement therapy at inclusion). We tested the positivity assumption by analysing cinacalcet use for different categories of PTH. There were both cinacalcet treated and non-treated in every PTH quintile both at baseline and during follow-up (Web Figure 3). Finally, we performed several sensitivity analyses including “time on treatment”, including more covariates and different model specifications (Web Tables 1–6). All analyses were performed using Stata 12 (StataCorp).

## Electronic supplementary material

Web Appendix
