# Supplementary material for: Cinacalcet use and the risk of cardiovascular events, fractures and mortality in chronic kidney disease patients with secondary hyperparathyroidism
Source: Sci Rep. 2018 Feb 1;8:2103. doi: 10.1038/s41598-018-20552-5 (PMC5794851; doi:10.1038/s41598-018-20552-5)
Supplement: Supplementary file 1 — Web Appendix [file 41598_2018_20552_MOESM1_ESM.pdf]

WEB APPENDIX

Cinacalcet use and the risk of cardiovascular events, fractures and mortality in chronic kidney disease patients with secondary hyperparathyroidism

*Marie Evans[1,2]

Shona Methven[1]

Alessandro Gasparini[5]

Peter Barany[1]

Kate Birnie[3]

Stephanie MacNeill[3]

Margaret T May[3]

Fergus J Caskey[1,3]

Juan-Jesus Carrero[4]

COMPLETE METHODS

Data sources

This study is based on the Stockholm CREAtinine Measurements (SCREAM) project(1), a healthcare-utilization cohort including all residents in the region of Stockholm, Sweden, undertaking at least one measurement of serum creatinine in inpatient or outpatient care during 2006–2011. Data were thereafter linked with regional and national administrative databases for information on healthcare utilization (International Classification of Diseases, Tenth Revision [ICD-10] codes and therapeutic procedures), complete information of drugs dispensed at Swedish pharmacies(2), validated referral to nephrology and renal replacement therapy endpoints(3) and vital status, with no loss to follow up.

Study population

We selected all individuals who had ever had a parathyroid hormone (PTH) test in Stockholm healthcare. Inclusion criteria were adult age (≥18 years) and attending secondary care nephrology with either an estimated glomerular filtration rate (eGFR) <60 ml/min/1.73m^2, a history of renal transplantation or undergoing maintenance dialysis therapy (ascertained by linkage with the Swedish Renal Registry(3)). Additionally, they had to have SHPT (defined as a PTH measurement at least twice above the upper reference limit, ≥130 ng/L); the date they met this criterion became their index date. Patients who had ever been prescribed cinacalcet before Jan 1 2006 were excluded (**Web Figure 1)**. The patients were followed from the index date until event, death, migration from the region, twelve months after the latest blood sample, or Dec 31 2012. The study protocol was approved by the ethical committee in Stockholm.

Exposure

The study exposure was cinacalcet treatment, as ascertained by the National Registry for Dispensed drugs which mandatorily records all dispensed prescriptions in Swedish pharmacies(2). Patients who were cinacalcet naïve at index date were followed monthly. Every month where no cinacalcet was dispensed at the pharmacy was recorded as "unexposed". The first pharmacy dispensation date of cinacalcet was used to determine date of treatment initiation and patient was recorded as "exposed" In the main analysis we applied an intention-to-treat (ITT) approach and assumed treatment to be maintained until event. In sensitivity analyses, we also analyzed the data "as-treated" that is, censoring patients three months after the last cinacalcet dispensation recorded.

Study covariates

Study covariates considered included age, sex, and a variety of laboratory tests, comorbidities and medications. All consecutive laboratory tests, performed in connection with encounters in primary care, outpatient specialist care or hospital care, were extracted from the three laboratories that provide services to the region (Aleris, Unilabs and Karolinska University Hospital laboratory). Inter- as well as intra-laboratory variation in laboratory measurements was considered minimal, as laboratories are frequently audited for quality and harmonization by the national organization EQUALIS (www.equalis.se). For this study we extracted all available laboratory results for plasma PTH, creatinine, phosphate, calcium, hemoglobin, albumin, c-reactive protein, and proteinuria (both dipstick and albumin/creatinine ratio). PTH was measured by four different methods at the three laboratories. The majority of the PTH measurements was analyzed by Roche Modular E170 (Karolinska, Unilabs), followed by Siemens Immulite 2000 XPi (Aleris), Abbott Architect i4000 (Aleris), and Siemens Advia Centaur XP (Unilabs). All three laboratories used second generation assays which also measured the PTH fragments and reported the results in ng/L. All serum creatinine measurements were standardized to isotope dilution mass spectrometry standards.

Glomerular filtration rate (eGFR) was estimated by the CKD-EPI equation(4). For patients receiving dialysis the eGFR was set at 2 ml/min/1.73m^2.

Comorbidities and surgical procedures were identified by ICD-10 codes issued in primary care health records, specialized out-patient care, and in-hospital care. We calculated co-morbidity history at the index date (history of diabetes, cardiovascular disease, hypertension, surgery for hyperparathyroidism, previous fracture, and a combined Charlson comorbidity index(5)). We updated the comorbidity information monthly during follow-up to capture new fractures and/or new surgery for hyperparathyroidism. The combined Charlson comorbidity index was updated every third month. Concurrent medication use considered all consecutive pharmacy purchases of angiotensin converting enzyme inhibitors (ACEi), angiotensin receptor blockers (ARB), beta blockers, calcium supplements, non-calcium containing phosphate binders, active vitamin D, nutritional vitamin D (inactive), erythropoiesis stimulating agents (ESA), and prednisolone or other steroids, which were all obtained from the National Registry for Dispensed drugs at index date and during follow-up. Patients were considered treated for three months after their last dispensation.

Study outcomes

The primary outcome was the composite of non-fatal and fatal cardiovascular events (hospitalization or death caused by myocardial infarction, angina pectoris, congestive heart failure, cerebrovascular disease, and peripheral arterial disease). In order to reduce the risk of misclassification, only main diagnoses were considered during follow-up for events..

Secondary outcomes were all-cause mortality and any new fracture during follow-up. Information regarding new fractures was obtained through linkages with in-patient and out-patient records.

Statistical analysis

The data were analyzed as per protocol, decided *a priori*, and based upon current knowledge of factors likely to influence clinical history and treatment decisions. Both time-fixed and time-dependent variables were included. The time-fixed variables were age, sex, history of diabetes, cardiovascular disease, hypertension, and previous surgery for hyperparathyroidism at index date.

Continuous time-dependent variables (hemoglobin, albumin, phosphate, creatinine, and eGFR) were categorized into quintiles of distribution. Due to markedly skewed distribution, PTH was log-transformed. The log-transformed variable showed satisfactory normality and was used as a categorical variable (quintiles). PTH assay was used as a categorical time-varying covariate for the four different types of analyses used. Medication, renal transplantation, dialysis treatment and Charlson comorbidity index were used as categorical, time-varying covariates.

The time-dependent covariates were organized into monthly measurements. If several laboratory measurements were available during a month, then the mean of them were used. If no new sample was recorded, we used the "last-value carried forward" principle. However, this was not applied if the period included the start date of cinacalcet treatment. Thus, all variables recorded at treatment initiation were measured *prior* to treatment start.

MSMs are needed in case a risk factor (e.g. PTH) both is associated with the chance of receiving the treatment and with the outcome, and at the same time the current levels of the risk factor are predicted by the past treatment (or non-treatment). When building the MSM careful consideration of the complete history of *all* possible and available variables over time is needed that may predict outcome and influence the doctor's decision to start or not to start the treatment; leaving important information out will lead to residual confounding. Thus, these models only work in large datasets, like ours, with rich information on covariates and

extensive data on follow-up. Other conditions also need to be fulfilled, one being the positivity assumption. This means that all patients at every level of the confounders included should have the possibility of being treated. To consider this criterion in our study we restricted to those who were under nephrology care and who had evidence of SHPT. Analyses further showed that there were both cinacalcet treated and non-treated in every PTH quintile both at baseline and during follow-up (Figure S2). We also considered the probability of being censored (i.e. the chance of not having developed the event by the end of follow-up) since this also affects the probability of treatment.

When constructing the MSM we started by calculating the stabilized inverse probability of treatment weights assuming the intercept was a smooth function using a natural cubic spline(6). The knots were placed at month 2, 11, 21, 37, and 63, which correspond to the 5, 27.5, 50, 72.5, and 95th centiles. We estimated the stabilized inverse probability weights of treatment using a logistic regression model which modelled the association of cinacalcet use with all time-fixed and time-varying covariates described above plus a time covariate until (and including) the month when treatment started(7). Only months with no missing values were included in the final analysis. We also included PTH, hemoglobin, calcium, phosphate, albumin, comorbidity index, dialysis status and treatment with active vitamin D three months before each month to account for increasing/decreasing time trends. We calculated the stabilized censoring weights similarly. However, we stipulated that variables twelve months before the current date predicted the risk of censoring. In the model for censoring weights we therefore included all time-fixed and time-varying covariates, and PTH, calcium, hemoglobin, albumin, comorbidity, dialysis status and active vitamin D treatment 12 months prior to each month to account for trends. In order to work well in the model, the stabilized weights should have a mean around 1.0 with low standard deviation (SD) and variance(6). Our stabilized treatment weights had a mean of 1.02 (SD 0.95, variance 0.91, range 0.009-

29.11). To reduce the risk of extreme weights affecting our results, we truncated the treatment weights at the 1,99th percentile (0.093, 2.73)(7) and used the truncated weights in all our main analyses. Our final weighted MSM included the stabilized treatment and censoring weights adjusting for past and current confounders of treatment. We present the results both with adjustment for baseline covariates in the final MSM (because the stabilized weights are conditional on baseline variables) and without. We also performed interaction tests for possible effect modification by some predetermined subgroups (age above and below 65 years, sex, history of diabetes and renal replacement therapy at inclusion).

Finally, we performed several sensitivity analyses: analyses were restricted to patients with a PTH increasing to more than twice the upper limit of normal (i.e. incident cases of SHPT) and patients with measured blood pressure at index date (including blood pressure in the treatment weights); and analyses were repeated based on exposure “as treated”, censoring patients 3 months after they discontinued treatment. . Furthermore, we tried different model specifications for constructing the treatment and censoring weights (PTH and other continuous variables modeled as a linear terms with three knot splines, and different levels of covariate adjustments). All analyses were performed using Stata 12 (StataCorp).

ICD-codes for classification of history of comorbidity and surgery

Diabetes: E10, E11

Cardiovascular disease: I21, I22, I23, I24, I25, I50, I61, I62, I63, I73, I461, I130, I132, I702, I739, I105, I115

Hypertension: I10, I11, I12, I13, I15

Surgery for Hyperparathyroidism: BBA30, BBA40, BBA50

Fracture: S02, S12, S22, S32, S42, S52, S62, S72, S82, S92

Renal transplantation: KAS10, KAS20 or registration in SRR (Swedish Renal Registry)

Charlson comorbidity index: We used the Stata command "charlson, (icd10)" to categorize the co-morbidities found in the National Patient Registry of ICD-10 codes(8).

ATC-codes for definition of drug purchases

Cinacalcet: H05BX01;

Vitamin D, active: A11CC03, A11CC04;

Vitamin D, inactive: A11CC05, A11AA01, A11JB, A12AX

Vitamin D, analogue: H05BX02;

Erythropoeitin stimulating agents: B03XA01, B03XA02, B03XA03;

ACEi/ARBs: C09A, C09B, C09C, C09D;

Betablockers: C07;

Calcium Supplements: A02AD01, A12AA04, A12AX, A12AA12, A12AA02;

Phosphate binders except calcium: V03AE04, V03AE02, V03AE03, A02AD01;

Prednisolone: H02AB01, H02AB06

ICD-codes for classification of outcomes

Cardiovascular event: Hospitalization with the main cause or cause of death being any of I21, I22, I23, I24, I25, I50, I61, I62, I63, I73, I461, I130, I132, I702, I739, I105, I115

Fracture event: Any hospital admission or patient contact coded with S02, S12, S22, S32, S42, S52, S62, S72, S82 or S92

Distribution of treatment weights for the main model

**Without truncation:**	Mean weights: 1.024, Standard deviation: 1.046, Variance: 1.09, Range 33.39-0.0956
**Truncation at 1,99th percentile**	Mean weights: 0.976, Standard deviation: 0.292, Variance: 0.085, Range 2.7649-0.096
**Truncation at treatment weights>10**	Mean weights: 1.00, Standard deviation: 0.565, Variance: 0.319, Range 9.99-0.096

Variables included estimating treatment weights

hx_hypertension, hx_diabetes, hx_fractures, hx_cardiovascular disease, hx_parathyroid suregery, age (years) as longitudinal*cubic, sex, s_creatinine at incl (cat), eGFR at inclusion (cat), s-PTH at inclusion (cat), hemoglobin at inclusion (cat), p-albumin at inclusion (cat), s-calcium at inclusion (cat), s-phosphate at inclusion (cat), ACEi/ARB treatment at inclusion, Betablocker treatment at inclusion, Vitamin D supplement at inclusion, active vitamin D at inclusion, Prednisolon at inclusion, ESA at inclusion, Calcium supplements at inclusion, Phosphate binders other than calcium at inclusion, Charlson comorbidity index score at inclusion, Renal replacement therapy at inclusion, PTH assay (cat), s-PTH follow-up (cat), hemoglobin follow-up (cat), p-albumin follow-up (cat), s-calcium follow-up (cat), s-phosphate follow-up (cat), ACEi/ARB treatment follow-up, Betablocker treatment follow-up, Vitamin D supplement follow-up, active vitamin D follow-up, Prednisolon follow-up, ESA follow-up, Calcium supplements follow-up, Phosphate binders other than calcium follow-up, Charlson comorbidity index score follow-up, Renal replacement therapy follow-up, Renal transplant follow-up, New fracture follow-up, s-PTH 3 months before, s-calcium 3 months before, s-phosphate 3 months before, hemoglobin 3 months before, p-albumin 3 months before, Charlson comorbidity index 3 months before, active vitamin D 3 months before, Renal replacement therapy 3 months before

Variables including for censoring weights

hx_hypertension, hx_diabetes, hx_fractures, hx_cardiovascular disease, hx_parathyroid suregery, age (years) as longitudinal*cubic, sex, s_creatinine at incl (cat), eGFR at inclusion (cat), s-PTH at inclusion (cat), hemoglobin at inclusion (cat), p-albumin at inclusion (cat), s-calcium at inclusion (cat), s-phosphate at inclusion (cat), ACEi/ARB treatment at inclusion, Betablocker treatment at inclusion, Vitamin D supplement at inclusion, active vitamin D at inclusion, Prednisolon at inclusion, ESA at inclusion, Calcium supplements at inclusion, Phosphate binders other than calcium at inclusion, Charlson comorbidity index score at inclusion, Renal replacement therapy at inclusion, PTH assay (cat), s-PTH follow-up (cat),

hemoglobin follow-up (cat), p-albumin follow-up (cat), s-calcium follow-up (cat), s-phosphate follow-up (cat), ACEi/ARB treatment follow-up, Betablocker treatment follow-up, Vitamin D supplement follow-up, active vitamin D follow-up, Prednisolon follow-up, ESA follow-up, Calcium supplements follow-up, Phosphate binders other than calcium follow-up, Charlson comorbidity index score follow-up, Renal replacement therapy follow-up, Renal transplant follow-up, New fracture follow-up, s-PTH 12 months before, s-calcium 12 months before, hemoglobin 12 months before, p-albumin 12 months before, Charlson comorbidity index 12 months before, active vitamin D 12 months before, Renal replacement therapy 12 months before

Variables included in the final weighted model (Model 2)

hx_diabetes, hx_fracture, hx_cardiovascular event, age categorized, sex, eGFR at inclusion (cat), s-PTH at inclusion (cat), hemoglobin at inclusion (cat), p-albumin at inclusion (cat), s-calcium at inclusion (cat), s-phosphate at inclusion (cat), ACEi/ARB treatment at inclusion, Betablocker treatment at inclusion, Vitamin D supplement at inclusion, active vitamin D at inclusion, Prednisolon at inclusion, ESA at inclusion, Calcium supplements at inclusion, Phosphate binders other than calcium at inclusion, Charlson comorbidity index score at inclusion

WEB TABLES

S1. Results after excluding 6 patients with extreme weights (no truncation)

Cardiovascular event*	OR 0.57; 95% CI: 0.37, 0.87
Mortality*	OR 0.77; 95% CI: 0.52, 1.15
Fractures*	OR 1.06; 95% CI: 0.52, 2.15

S2. Results after truncating treatment weights at >10

Cardiovascular event	OR 0.57; 95% CI: 0.37, 0.86
Mortality	OR 0.76 95% CI: 0.52, 1.12
Fracture	OR 1.10; 95% CI: 0.60, 2.04

S3. Results for primary outcome in a subsample (n=3082) with measured blood pressure

Cardiovascular event*	OR 0.56; 95% CI: 0.33, 0.97
Cardiovascular event#	OR 0.80; 95% CI 0.56; 1.15
Mortality#	OR 0.79; 95% CI: 0.55, 1.12
Fracture#	OR 1.16; 95% CI: 0.63, 2.14

S4. Results for analyses with time "on treatment" (the number of events for the primary outcome dropped to 1295 among non-treated and 155 among treated)

Cardiovascular event#	OR 0.77; 95% CI: 0.53, 1.14
Mortality#	OR 0.86; 95% CI: 0.57, 1.31
Fracture#	OR 1.14; 95% CI: 0.50, 2.63

S5. Results for those with incident PTH >X2 (n=2958)

Cardiovascular event#	OR 0.77; 95% CI: 0.51, 1.17
Mortality#	OR 0.78; 95% CI: 0.56, 1.10
Fracture#	OR 0.97; 95% CI: 0.50, 1.88

#model with truncation at 1,99th percentile and adjustment for baseline variables in the MSM

*model without

S6. Results for primary outcome (cardiovascular event) with different model specifications for determination of stabilizing weights.

	Mean weights/Truncated 1,99th percentile mean of weights	Range (SD)/ Truncated 1,99th percentile Range (SD)	OR (95% CI)	OR (95% CI) with truncated weights at 1,99th percentile
Denominator with BL+ PTH_0 & PTH_{TV} in categories and time. Numerator with PTH_0 + BL + time	1.00/0.98	0.069 – 20.74 (0.63) / 0.15 – 2.83 (0.29)	0.54 (0.34-0.86)	0.68 (0.49 – 0.93)
Denominator and numerator as above but PTH_0 & PTH_{TV} were replaced by a linear term with three know spline	1.05/0.99	0.020 – 50.08 (1.41) / 0.07 – 3.89 (0.41)	0.41 (0.25 – 0.69)	0.54 (0.37 – 0.79)
Denominator and numerator as specification 2, adding baseline and time-varying calcium, phosphate, RRT, TX and Charlson index. Lab values linear with three-knot spline.	1.06/0.98	0.001 – 63.05 (1.72) / 0.06- 3.62 (0.37)	0.45 (0.25 – 0.79)	0.59 (0.40 – 0.86)
Denominator and numerator as specification 2, adding baseline and time-varying calcium, phosphate, RRT, TX + Medication. Lab values linear with three-knot spline.	1.06/0.98	0.001 – 83.79 (2.08) / 0.06 – 3.30 (0.35)	0.48 (0.28 – 0.84)	0.60 (0.41 – 0.88)

BL: All models included a linear*cubic term for age, sex, RRT at baseline, diabetes, cardiovascular disease and history of fractures at baseline, and combined linear Charlson comorbidity index at baseline

Table S7. Baseline characteristics in a cohort of 3,526 referred patients with chronic kidney disease and first parathyroid hormone >130 ng/L, 2006-2011 in the region of Stockholm, Sweden

**Characteristic**	**Variable, if missing data [n]**	
	Age, median years	66.8 (54.3-75.7)
	Men	2,277 (64.6)
**Comorbidity**		
	Hypertension	1,605 (45.5)
	Cardiovascular disease	1,480 (42.0)
	Diabetes mellitus	761 (21.6)
	Charlson comorbidity index, mean	3.92 (2.1)
	Previous surgery for hyperparathyroidism	50 (1.4)
	Previous fracture	449 (12.7)
**Laboratory values**		
	Parathyroid hormone, median	183 (150 – 265)
	S-creatinine, median [3483]	267 (196 – 434)
	Hemoglobin [3437]	119.7 (16.9)
	P-Albumin, median [3433]	35 (32 – 38)
	P-Calcium [3451]	2.26 (0 .18)
	P-phosphate, median[3429]	1.4 (1.1 – 1.6)
	Albuminuria [2,157]	
	none	672 (31.1)
	micro	388 (18.0)
	macro	1,097 (50.9)
	eGFR, median [3483]	18.6 (9.4 – 27.1)
**Medication**		
	ACEi or ARB	2,509 (71.2)
	Betablocker	2,257 (64.1)
	Vitamin D (active)	1,636 (46.4)
	Vitamin D supplement	206 (5.8)
	Erythropoesis stimulating agents	1,121 (31.8)
	Calcium supplement	1,132 (32.1)
	Phosphate binder use	476 (13.5)
	Prednisolone	876 (24.8)
**Renal replacement therapy**		
	Dialysis	607 (17.2)
	Renal transplantation	415 (11.8)

All continuous values are presented as means and standard deviation (SD) unless indicated. Categorical values are presented as number (percentage, %). Parathyroid hormone (ng/L=pg/mL), S-creatinine (µmol/l), Hemoglobin (g/L), P-albumin (g/L), P-calcium (mmol/L), P-phosphate (mmol/L), eGFR (glomerular filtration rate estimated by CKD-EPI equation) in ml/min/1.73m^2, ACEi (Angiotensin Converting Enzyme inhibitor), ARB (Angiotensin Receptor Blocker)

Table S8. Comparison of characteristics between our study cohort and the EVOLVE study at the time of cinacalcet initiation

		**No Cinacalcet** (n=3091)**	**New Cinacalect use (n=435)**	**EVOLVE Placebo (n=1935)**	**EVOLVE Cinacalcet (n=1948)**
**Characteristic**					
	Age, median years (P_{10}-P_{90})	67.7 (56-77)	62.6 (41-79)	54 (35-73)	55 (35-74)
	Men	65.5	57.9	60.3	58.5
**Comorbidity**					
	Diabetes mellitus	21.8	19.8	33.6	33.5
	Heart failure	34.3	28.7	23.6	23.1
	Peripheral vascular disease	17.3	14.0	16.6	16.1
	Myocardial infarction	26.4	18.6	12.6	12.3
	Stroke	16.4	10.6	8.3	10.0
	Previous surgery for hyperparathyroidism	1.4	1.4	4.5	4.7
	Previous fracture	12.9	11.3	20.0	19.6
**Laboratory values**	Parathyroid hormone, median	182 (126-390)	636 (290-1150)	690.0 (363.0, 1683.0)	694.5 (362.0, 1707.0)
	P-Phosphate, median mg/dl	4.0 (2.8-6.3)	5.6 (3.7-8.1)	6.2 (4.9, 8.4)	6.3 (4.9, 8.3)
	B-Haemoglobin g/dl	11.9	11.9	11.8	11.7
	P-Albumin, median d/dl	3.5 (2.7-4.0)	3.4 (2.8-3.9)	3.7 (3.2, 4.1)	3.7 (3.2, 4.1)
	P-Calcium, median mg/dL	9.12 (8.3-9.8)	9.8 (8.7-10.8)	9.8 (9.0, 10.7)	9.8 (9.0, 10.7)
**Medication**	Use of ACEi or ARB	73.4	65.1	42.7	44.8
	Use of Beta-blocker	71.4	61.3	48.5	45.4
	Use of Vitamin D (active)	69.8	72.6	58.1	58.3
	Use of Vitamin D supplement (inactive)	7.5	3.2	3.3	2.6
	Use of erythropoiesis stimulating agents	43.2	48.7	85.7	84.2
	Use of Calcium binder	42.8	45.1	53.0	53.2
	Use of Phosphate binder without calcium	24.3	72.6	36.0	34.6

**Renal replacement therapy**	Dialysis	20.0	62.5	100	100
	Renal transplantation	14.5	12.4	0	0

**For those who were *not* prescribed cinacalcet we calculated a fictive date during follow-up at 23 months after index date, and for those who did not have such long follow-up the latest recorded variables. Continuous variables presented as median, (P_{10}-P_{90}).

SUBGROUP ANALYSES

We tested the interaction by several predefined subgroups. In order to avoid multiple testing we decided not to perform subgroup analyses unless the initial test for interaction was positive. Below are the results from these interaction tests (model with truncation and without adjustment for baseline variables in the MSM model).

Interaction between Cardiovascular event and

Female sex	OR 0.92; 95% CI 0.50, 1.69	p=0.79
Age >65 years	OR 1.14; 95% CI 0.63, 2.01	p=0.66
Diabetes	OR 1.31, 95% CI 0.63, 2.73	p=0.47
RRT	OR 1.25, 95% CI 0.68, 2.28	p=0.47

Interaction between All-cause mortality and

Female sex	OR 1.08; 95% CI 0.81, 1.07	p=0.35
Age >65 years	OR 0.70; 95% CI 0.40, 1.26	p=0.24
Diabetes	OR 1.47; 95% CI 0.73, 2.96	p=0.28
RRT	OR 1.43; 95% CI 0.75, 2.73	p=0.28

Interaction between Fractures and

Female sex	OR 1.30; 95% CI 0.45, 3.80	p=0.63
Age >65 years	OR 1.87; 95% CI 0.62, 5.65	p=0.27
Diabetes	OR 0.39; 95% CI 0.13, 1.15	p=0.09
RRT	OR 1.72; 95% CI 0.49, 6.10	p=0.40

Odds Ratio for Fracture for cinacalcet users with diabetes

OR 0.42; 95% CI 0.15, 1.19 p=0.10

Odds Ratio for Fracture for cinacalcet users without diabetes

OR 1.40 95% CI 0.78, 2.51 p=0.53

SUBGROUPS BY OUTCOME

Hospitalization cardiovascular event	OR 0.67; 95% CI 0.47; 0.95
Hospitalization for Heart Failure	OR 0.56; 95% CI 0.33, 0.97
Hospitalization for Ischemic heart disease	OR 0.55; 95% CI 0.33, 0.93
Hospitalization for Stroke	OR 0.47; 95% CI 0.17, 1.34

WEBFIGURES

Figure S1. Time-dependent Cox proportional hazards regression of the primary outcome, adjusted for variables used in the treatment model but without adjustment for time-dependent confounding by IPW

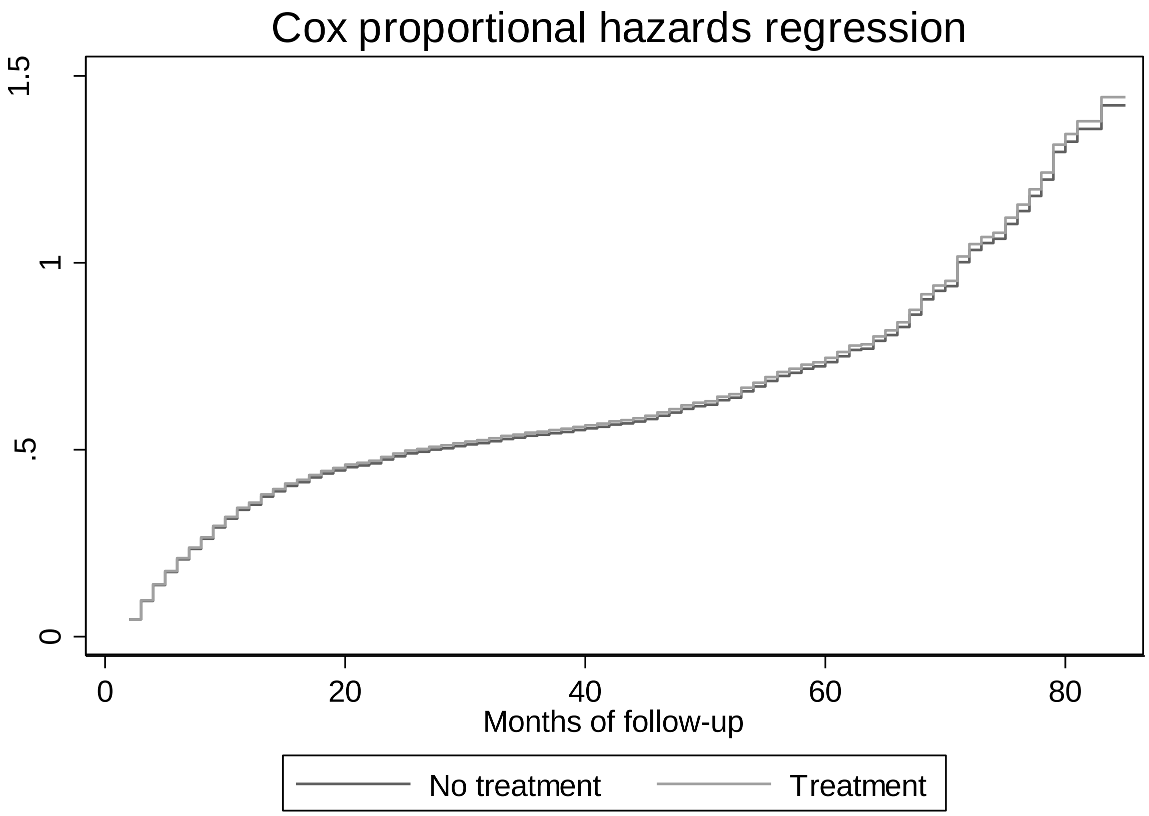

Figure S2. Patient's flow chart

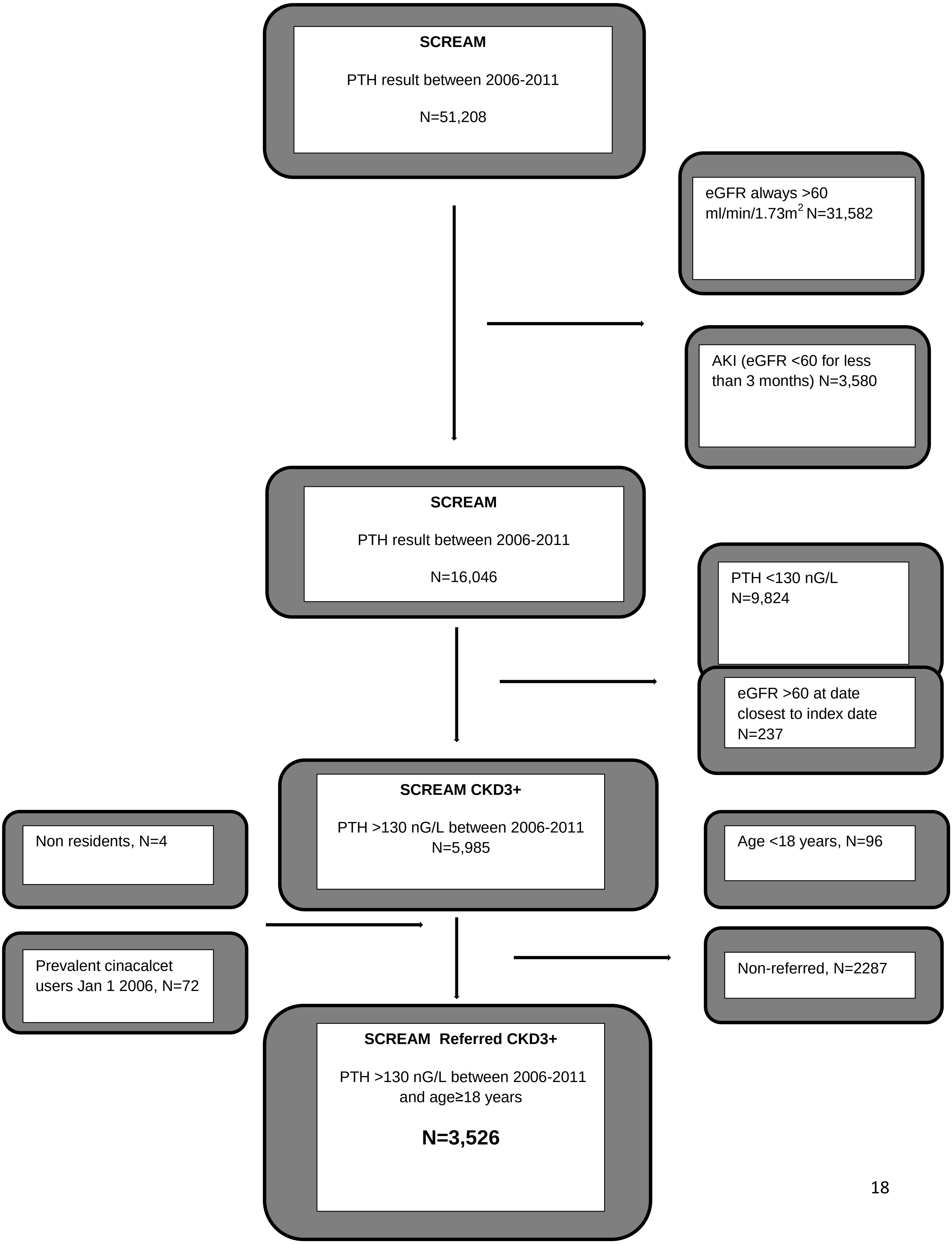

Figure S3. Distribution of S-PTH values in Cinacalcet treated and non-treated over the entire follow-up period

A.

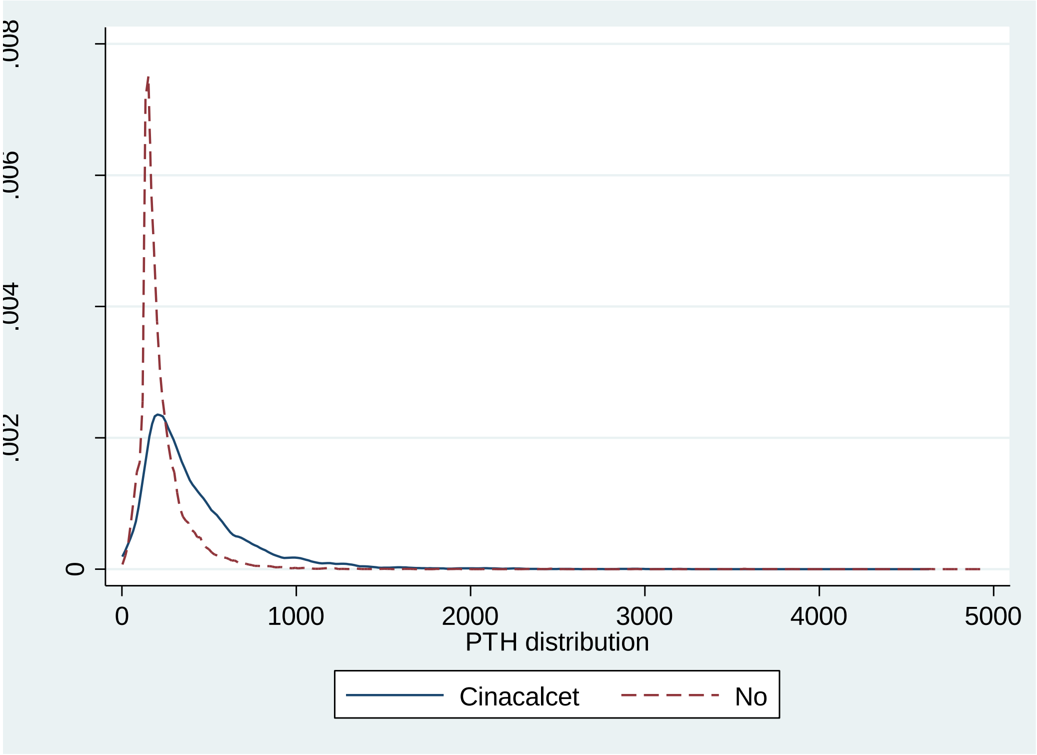

B.

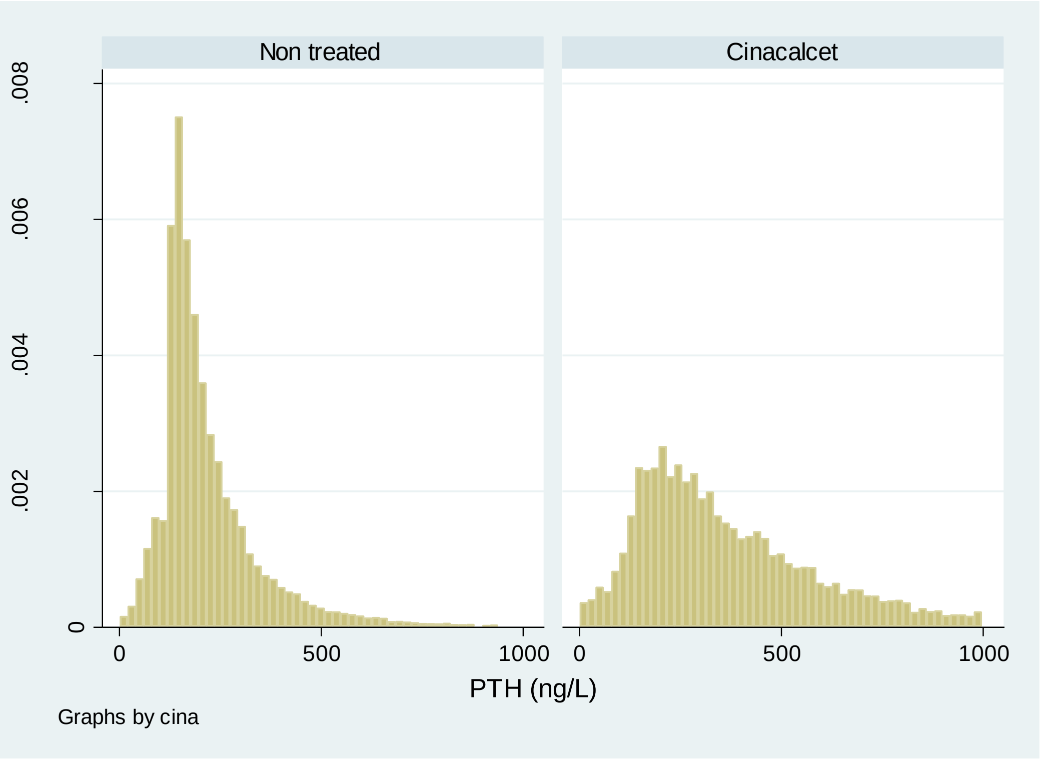

REFERENCES

1. Runesson B, Gasparini A, Qureshi AR et al. The Stockholm CREAtinine Measurements (SCREAM) project: protocol overview and regional representativeness. Clinical Kidney Journal 2016;9:119-127.
2. Wettermark B, Zoega H, Furu K et al. The Nordic prescription databases as a resource for pharmacoepidemiological research--a literature review. Pharmacoepidemiol Drug Saf 2013;22:691-9.
3. Swedish Renal Registry. Swedish Renal Registry: Annual report. www.snronline.se ed: www.snronline.se, 2014:1-52.
4. Levey AS, Stevens LA, Schmid CH et al. A New Equation to Estimate Glomerular Filtration Rate. Ann Intern Med 2009;150:604-612.
5. Charlson M, Pompei P, Ales K, McKenzie C. A new method of classifying prognostic comorbidity in longitudinal studies: development and validation. J Chron Dis 1987;40:373-383.
6. Fewell Z, Hérnan MA, Wolfe F, Tilling K, Choi HK, Sterne JA. Controlling for time-dependent confounding using marginal structural models. the Stata Journal 2004;4:402-420.
7. Cole SR, Hernán MA. Constructing Inverse probability weights for marginal structural models. Practice of Epidemiology 2008;168:656-664.
8. Quan H, Sundararajan V, Halfon P, et al. Coding algorithms for defining comorbidities in ICD-9-CM and ICD-10 administrative data. Medical Care 2005;43:1073-1077.
